# Surface ocean metabarcoding confirms limited diversity in planktonic foraminifera but reveals unknown hyper-abundant lineages

**DOI:** 10.1038/s41598-018-20833-z

**Published:** 2018-02-07

**Authors:** Raphaël Morard, Marie-José Garet-Delmas, Frédéric Mahé, Sarah Romac, Julie Poulain, Michal Kucera, Colomban de Vargas

**Affiliations:** 1 MARUM Center for Marine Environmental Sciences, University of Bremen, Leobener Strasse, 28359 Bremen, Germany; 2 Station Biologique de Roscoff, UMR7144, Sorbonne Universités UPMC Université Paris 06 & CNRS, Place Georges Teissier, 29680 Roscoff, France; 3 CIRAD, UMR LSTM, F-34398 Montpellier, France; 4 CEA—GENOSCOPE- Institut François Jacob, 2 rue Gaston Crémieux, 91057 Evry, France

## Abstract

Since the advent of DNA metabarcoding surveys, the planktonic realm is considered a treasure trove of diversity, inhabited by a small number of abundant taxa, and a hugely diverse and taxonomically uncharacterized consortium of rare species. Here we assess if the apparent underestimation of plankton diversity applies universally. We target planktonic foraminifera, a group of protists whose known morphological diversity is limited, taxonomically resolved and linked to ribosomal DNA barcodes. We generated a pyrosequencing dataset of ~100,000 partial 18S rRNA foraminiferal sequences from 32 size fractioned photic-zone plankton samples collected at 8 stations in the Indian and Atlantic Oceans during the *Tara* Oceans expedition (2009–2012). We identified 69 genetic types belonging to 41 morphotaxa in our metabarcoding dataset. The diversity saturated at local and regional scale as well as in the three size fractions and the two depths sampled indicating that the diversity of foraminifera is modest and finite. The large majority of the newly discovered lineages occur in the small size fraction, neglected by classical taxonomy. These unknown lineages dominate the bulk [>0.8 µm] size fraction, implying that a considerable part of the planktonic foraminifera community biomass has its origin in unknown lineages.

## Introduction

After ~250 years of Linnean taxonomic work, >90% of the ocean’s biodiversity still appears to be undescribed^1^. The quest to obtain an inventory of marine species is hampered by the difficulty to exhaustively sample the vast three-dimensional oceanic habitat. Because new proposals for species description require re-evaluation of existing knowledge^2^, the taxonomic effort required to describe the unknown diversity increases as the Linnaean catalogue becomes more complete. At the current pace, the completion of the Linnaean catalogue seems to be a never-ending task, precluding an understanding of the function of the presumably unknown 90% of marine biodiversity.

A response to these limitations is to rely on targeted high throughput sequencing of environmental samples, or metabarcoding. Metabarcoding attempts to obtain an exhaustive inventory of all organisms in a given sample by sequencing short informative DNA barcodes (typically fragments of ribosomal genes). The obtained DNA sequences are clustered into Molecular Operational Taxonomic Units (MOTUs) which can be seen as proxies for the presence and relative abundance of the taxa present in the samples^2^ although the actual relative abundance can be biased by differential rDNA copies number across organisms^3^ and diversity estimates can be inflated because of intragenomic polymorphisms^4^. No time-consuming sorting of single specimen and expert taxonomic assessment is involved in the process, but the actual organisms are never directly observed. Instead, the link with existing taxonomic knowledge is made by the comparison of the MOTUs with curated reference databases^5,6^, mostly derived from barcoding efforts^7^. The taxonomic interpretation of environmental MOTUs relies on the completeness of the existing reference databases and the taxonomic resolution of the chosen barcodes.

So far, metagenomic surveys of marine biodiversity depict the oceanic realm as a treasure trove of diversity^8^. Microbial marine communities seem to be composed of a small number of abundant taxa co-occurring with hugely diverse, undescribed and rare consortia. This “rare biosphere”^8^ is universally observed amongst planktonic^9,10^ and benthic^11^ communities. Indeed, the recent metabarcoding survey of sunlit ocean by the *Tara* Oceans project revealed that cosmopolitan MOTUs, representing 0.35% of the diversity, accounted for 68% of the volume of the dataset^10^. Less than 1% of the detected MOTUs had a perfect match with a reference sequence. This suggests that a large portion of the marine biosphere is still uncharacterized by DNA barcoding.

However, metabarcode diversity can be inflated by the presence of chimeric sequences generated during PCR-based amplification^12^, or by sequencing errors^13^. Stringent quality control and detection algorithm such as UCHIME^14^ are commonly used to remove potential chimeras from the dataset. But this practice bears the risk of removing genuinely rare sequences from the dataset and thus “throw the baby out with the bathwater”^15^. Parsing the ecological signal from the noise in metagenomic datasets is challenging^16^ and mostly study-dependent, which impedes effort to compare different datasets that could reveal informative fine-scales community structures^17^. Modeling approaches^18^, mock communities^16^, and application of ecological metrics^15^ can help to assess or reduce the impact of false and low abundance MOTUs, but ultimately the genuine species richness of an ecosystem investigated with metabarcoding approach is debatable^19^.

In summary, the availability of novel sequencing technologies brings new challenges in the assessment of biodiversity. Such assessment requires the simultaneous availability of curated reference databases, well-resolved barcodes, stringent but flexible bioinformatics pipelines and sufficient background taxonomic knowledge. In this study, we bring these elements together and use planktonic foraminifera, arguably the best-known group of pelagic protists, as a case study to assess the extent of their diversity through metabarcoding survey.

Planktonic foraminifera is a group of ubiquitous pelagic marine protists with reticulated pseudopods secreting a calcareous shell and clustering within the Rhizaria^20^. The Foraminifera appeared in fossil record in the early Cambrian^21^ but colonized the plankton only in the toarcian^22^. Plankton has been invaded several times by independant lineages of benthic foraminifera^23,24^ and has a result, the planktonic foraminifera are polyphyletic^25^. Since their first systematic description by d’Orbigny (1826), planktonic foraminifera have been a cornerstone of marine sediments dating^26^ and paleoceanographic reconstructions^27^. Their global geographic distribution, seasonal dynamics and trophic behavior have been studied by sampling in the plankton^28^, sediment traps^29^ and surface sediments^30^. After two centuries of taxonomic investigations, extant planktonic foraminifera diversity seems to have settled with ~50 morphologically defined species^31^, much less than the 600–800 extant species of radiolaria^32^, the sister group of foraminifera within the Rhizaria. This traditional view of the diversity in planktonic foraminifera has been challenged by the discovery of cryptic diversity manifested in rRNA sequences^33^. It revealed that most modern morpho-species of planktonic foraminifera are aggregates of cryptic biological species^34^. This unique single-cell sampling and rRNA sequencing effort has resulted in the creation of a comprehensive reference database, which includes 3,322 single-cell sequences of major morphospecies producing >90% of the recent fossil assemblages^35^ with curated taxonomy at the level of morphological and cryptic (biological) species.

In the present study, we confront these two centuries of classical taxonomy, followed by two decades of single-cell genomics with a metabarcoding approach. We explore the planktonic foraminifera diversity in 32 size-fractioned samples collected at 8 localities representing the Indo-Pacific and Atlantic biomes sampled during the *TARA Oceans* expedition^36^. These samples were used to generate foraminifera-specific metabarcodes by enriching the DNA extract through PCR amplification of a well-constrained foraminiferal barcode. The metabarcode was sequenced with Roche/454 pyrosequencing as it offers longer reads and thus more phylogenetic information to interpret potential novel lineages. The obtained barcodes were structured into MOTUs and integrated into existing molecular taxonomic framework^33^ to assess the extent of planktonic foraminifera diversity.

## Material and Methods

### Sampling of total plankton and amplification/sequencing of foraminiferal DNA

Plankton samples were recovered during the *Tara* ocean circum-global expedition^36^, from water volumes allowing theoretical saturation of plankton biodiversity in each organismal size-fraction^37^. We selected 32 samples collected at 8 stations, 2 depths (Surface, between 0 and 10 meters, and Deep Chlorophyll Maximum, between 50 and 120 meters) and from 3 different plankton size fractions: micro- [20–180 µm], meso- [180–2000 µm] and bulk [>0.8 µm] plankton (Supplementary Material 1) the filtered volume of water varied between 90 liters for the smallest sized fraction to 736 m³ for the largest mesh size. The 8 selected *Tara* Oceans stations represented a mosaic of oceanic conditions: Indian Ocean (stations 64 and 65), Benguela current (stations 66 and 67), Agulhas rings (stations 68 and 78) and Sub-tropical Atlantic Ocean (stations 70 and 76)^37^. All the selected samples have already analyzed using the V9 universal barcode primers and presented in^10^. The details of sample collection, preservation and DNA extraction are presented in this study. Here, to amplify the relatively rare foraminiferal rDNA fragments out of plankton DNA extracts we developed a specific semi-nested PCR protocol. The first PCR amplification was carried out by mixing 1 µl of DNA extract with 0.4 µM of each specific foraminiferal primers S14F1 (5′-CCATCTCATCCCTGCGTGTCTCCGAC-3′) and S19F (5′-GTACRAGGCATTCCTRGTT-3′)^38,39^, 6% ethylene Glycol (Fisher BP 230.1), 3% of DMSO and 1X Mix Phusion High Fidelity DNA polymerase (Thermo Scientific F 532L) in a final volume of 25 µl. PCR amplification conditions were as follows: initial denaturation at 98 °C for 60 seconds followed by 11 cycles at 98 °C for 10 seconds, 55 °C for 30 seconds and 72 °C for 30 seconds, and 11 cycles at 98 °C for 10 seconds, 52 °C for 30 seconds and 72 °C for 30 seconds and 10 minutes of final extension at 72 °C. The reverse S19F primer was modified with an adaptor of 30 nucleotides, a key of 4 nucleotides and a tag of 8 nucleotides appended to the PCR primer for Roche/454 sequencing. Each of the 32 resulting PCR products was subjected to a second round of amplification using the couple S15rF (5′-GTGCATGGCCGTTCTTAGTTC-3′)^39^ and S19F. Thirty-two unique tagged S15rF primers were designed for Roche/454 sequencing to be unique, with a minimum of two differences between any pair of tags, and no more than 2 consecutive identical nucleotides (OBItools, http://metabarcoding.org/obitools) and to avoid the formation of strong secondary structures with the Roche/454 adaptor A or the forward primer S15rF. The second PCR consisted in 1 µL of PCR product of the first PCR mixed with 0.4 µM of each tagged foraminifer-specific primers, 3% of DMSO and 1X Mix Phusion High Fidelity DNA polymerase (Thermo Scientific F 532L) in a final volume of 25 µL, with the following amplification conditions: initial denaturation at 98 °C for 60 seconds followed by 25 cycles at 98 °C for 10 seconds, 53 °C for 30 seconds and 72 °C for 30 seconds and 10 minutes of final extension at 72 °C. Four PCR replicates and one negative control per samples were run in the second amplification to obtain enough DNA. Replicate PCR products were pooled for purification using the NucleoSpin® PCR Clean-Up (Macherey-Nagel) kit, and eluted in 20 µL of buffer following the manufacturer’s instructions. The concentration of PCR products were quantified with PicoGreen double strain DNA Quant-i^T^ TM Kit (Invitrogen) with the Safire2 (TECAN). PCR products were mixed in equal concentration to obtain a similar number of amplicons per sample and were sequenced with the Roche/454 GS-FLX Titanium pyrosequencing technology (Genoscope, Paris).

### Filtering and clustering of 454 pyrosequencing rDNA amplicons

Only the amplicons containing the exact forward primer and no ambiguous nucleotide were retained. Potential chimeras were discarded using the UCHIME implementation in USEARCH 7.0 with default parameters^14^. Because of the relative long length of the PCR product sequenced, ranging from 350 to 700 bp, only 32.22% of the sequences reached the reverse primer. Therefore, the sequences were trimmed off at 300 bp after the forward primer and clustered using SWARM v1.2.8^40^ using a local threshold of two differences (*d* = 2) for accommodating the extreme rate of rDNA substitution known in planktonic foraminifera^41^. The obtained MOTUs were assigned to their closest hit in the *Protist Ribosomal Reference* database (*PR2*, based on GenBank v. 201)^5^, also truncated at 300 bp after the forward primer, using ggsearch as implemented in FASTA v36.3.5c^42^. The result of the attribution is given in Supplementary Material 1. The raw sequence data can be downloaded from the European Nucleotide Archive under BioProject PRJEB23355 (https://www.ebi.ac.uk/ena/data/view/PRJEB23355).

### Phylogenetics

We used a conservative and strict sequence selection process to analyze the phylogenetic structure of our dataset. In a conservative approach, only MOTUs attributed to foraminifera (Level 3 of PR² database ranked taxonomy), occurring in at least three samples and having a total abundance of at least ten reads were first considered for downstream analysis (Supplementary Fig. 1). The most abundant sequence of each MOTU was used as a prefix to search in the metabarcoding dataset the longest sequences of each MOTU that could have potentially reached the reverse primer. We retrieved the most abundant of the longest sequence of each of the retained MOTU to use them as a MOTU “representative sequence”. The representative sequences were manually checked, and those having an insufficient length or being potential chimera possibly omitted by UCHIME were excluded from the analysis.

**Figure 1:**
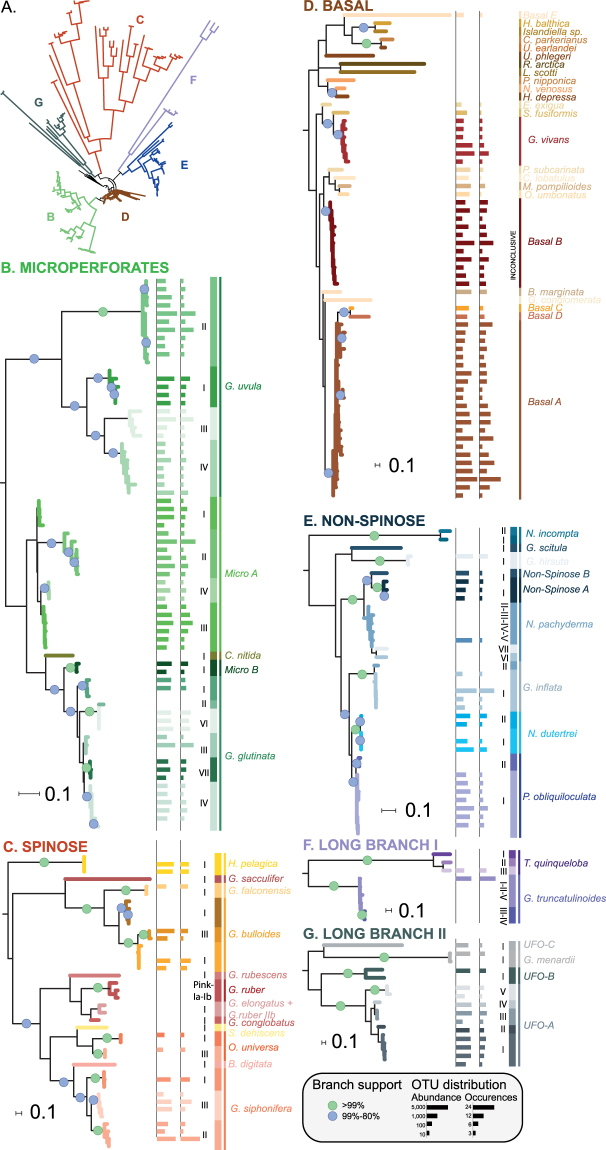
(**A**) Maximum Likelihood phylogenetic inference for planktonic foraminifera environmental and reference sequences. The tree, rooted on Textulariida sequences, includes 81 reference sequences of benthic and planktonic foraminifera, together with 155 representative sequences of each retained environmental MOTUs. The colored branches highlight the position of the major clades in the tree. (**B**–**F**) Individual clades shown in details. The branch support is highlighted by dots on the branch. The Bar chart on the right panel shows the abundance and occurrence of each MOTU in the dataset (Log scale). The two ranks ABGD species delimitation is provided by the vertical bars at the extreme right of each panel with the associated names next to it. The colors of the branch correspond to the “Genetic type” level delimitation, except for the “basal” clade were only the morphological level is considered.

The retained representative sequences were automatically aligned using MAFFT v.7^43^ with 81 sequences representative of foraminifera diversity. We selected 57 planktonic foraminiferal reference sequences representing the morphospecies with an existing barcode with their sub-division into genetic type (or cryptic species) derived from the *Planktonic Foraminifera Ribosomal Reference* database (*PFR²*)^35^, and 24 representative sequences of the major groups of benthic foraminifera with multi-locus wall chambered tests (Globothalamea)^44^. The best substitution model was selected using jModeltest v. 2^45^ and a phylogenetic inference was carried out using PhyML^46^ with 1,000 bootstrap pseudo replicates for estimation of the branch support. The resulting tree was visualized with iTOL^47^ (Fig. 1). The alignment and resulting tree inference are provided in Supplementary Material 2.

### A molecular nomenclature framework for planktonic foraminifers

No unique rDNA dissimilarity threshold exists to discriminate genuine biological species in foraminifera^33,48–50^, or other group of protists^51–53^. The availability of the PFR² database permits to produce a flexible framework that do not rely on fixed threshold but on the extensive taxonomic knowledge produced through single-cell genetic studies and to extend its properties onto the environmental sequences. To parse the different level of diversity of the environmental dataset into meaningful units, we produced a 4-rank molecular nomenclature taxonomic framework (Morphogroup|Genus|Morphospecies|Genetic Type) harmonized between the sequences derived from the reference database and the MOTUs representative sequences. To this end, we applied the Automated Barcode Gap Discovery algorithm (ABGD)^54^ on crown groups of closely-related sequences displayed on the Fig. 1A: Basal, Microperforates, Non-Spinose, Spinose, Long-Branch I and Long Branch II (See results for description of the groups). ABGD was run using the K80 distance option with a relative distance gap of 0.5 with 100 consecutive steps to cluster progressively the reference and environmental sequences from the level representing genetic types^50^ to the level representing morphological species. We used the existing delineation at both genetic type and morpho-species levels among the reference sequences^35^ to select the levels of genetic and morphological taxonomy amongst the MOTUs plateaus proposed by ABGD. MOTU plateau corresponding to genetic types were defined as the lowest plateau not merging reference sequences from distinct genetic lineages of the same morphospecies. Likewise, MOTU plateau corresponding to morpho-species were defined as the lowest plateau not merging reference sequences from distinct morphospecies. The robustness of the ABGD-based delineation at the two taxonomic levels was then evaluated with patristic distances calculated on the phylogenetic tree^55^ (Supplementary Fig. 2) coupled with Kolmogorov-Smirnov and Mann-Whitney tests calculated with PAST 2.17^56^ for distance comparisons (Supplementary Table 1).

### Assignation of MOTUs to meta-reference database for ecological analyses

**Figure 2:**
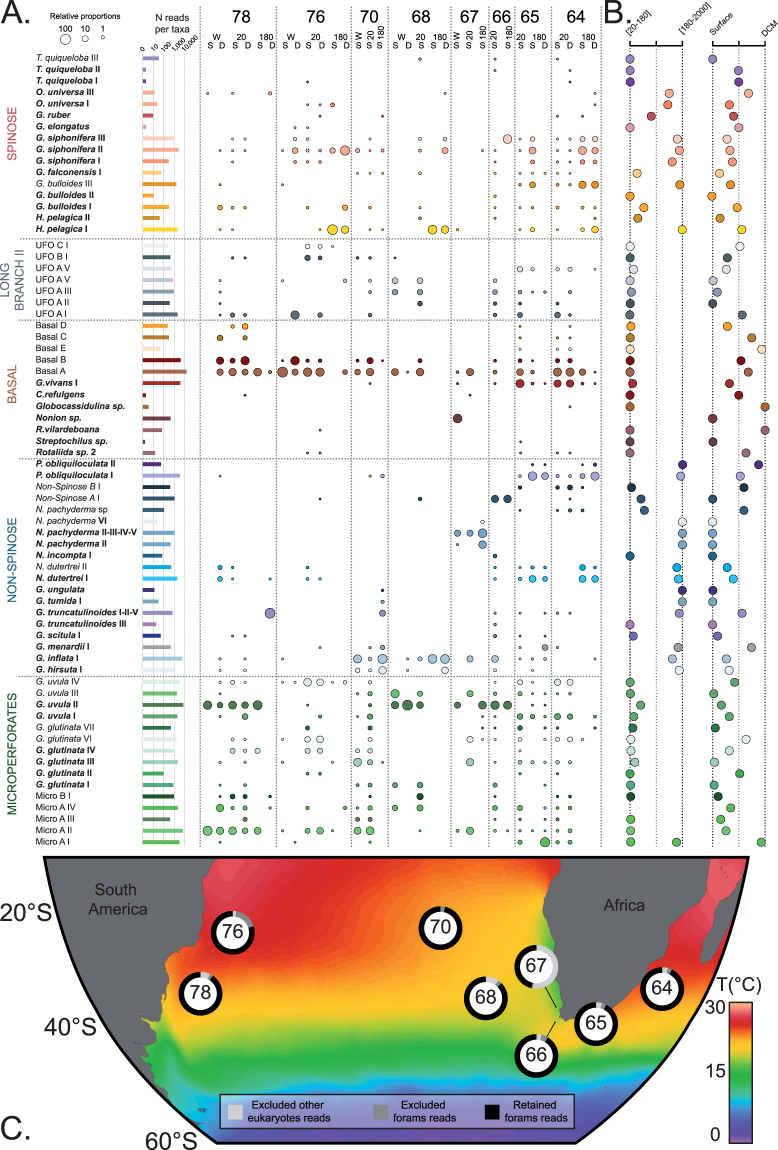
Ecological dataset. (**A**) Abundance and occurrence of the 69 planktonic foraminiferal taxa detected in our environmental survey. The lines represent the occurrence of each taxon in all explored Tara Oceans stations, and the columns represent the individual samples. The size of the circles is proportional the relative abundance of each taxon within each sample (log values). 180, 20 and W correspond to the [180–2000 µm], [20–180 µm] and [>0.8 µm] size fractions. D and S correspond to the Deep Chlorophyll Maximum and Surface depths, respectively. (**B**) Relative occurrences of each taxon between the size fractions [20–180 µm] and [180–2000 µm] and between surface and DCM. (**C**) Geographic location of the station collections. The ring around the station number shows the partition of the reads obtained at each station. The background shows the mean annual Sea Surface Temperature extracted from the World Ocean Atlas 2013^65^ and was generated with Ocean Data View^66^.

**Figure 3:**
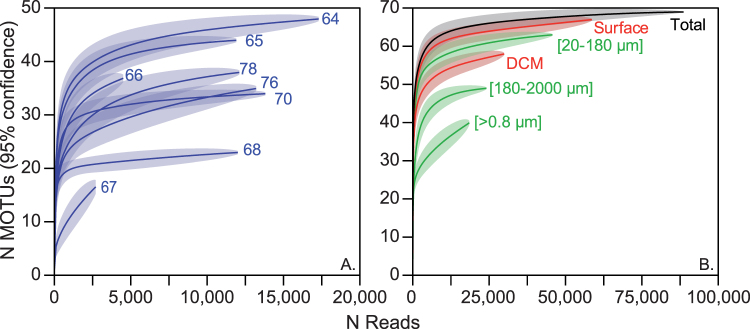
Rarefaction curves of foraminifers based on the ecological dataset (Fig. 2) for the sampling stations (**A**) and for the organismal size fractions, sampling depth and overall dataset (**B**).

The resulting 4-level ranked molecular nomenclature produced by the hierarchal ABGD clustering allowed re-assigning all the rare MOTUs (occurring in less than 3 samples and with less than 10 reads) and potential variants of abundant MOTUs, which were initially not considered in our analysis. To incorporate the maximum of the dataset in the final analysis, we produced a *meta-*reference database that included the *PFR²* database, the benthic foraminifera reference sequences available in the *PR²* database and the representative sequences of the environmental MOTUs. We aligned the taxonomic framework of the PR² and PFR² on the 4-level ranked nomenclature (Supplementary Material 3) and reassigned all MOTUs with the meta reference database (Supplementary Fig. 3, Supplementary Material 1). All MOTUs having an identity greater than 95% with sequences in the *meta-reference* and without ambiguous attribution were retained (Supplementary Fig. 3, Supplementary Material 1) and merged to produce the final ecological dataset (Fig. 2, Supplementary Material 4). MOTUs rarefaction curves at each *Tara* Oceans station, depth of collection, within each plankton size fraction, and for the total dataset were inferred using PAST v 2.17^56^ (Fig. 3).

## Results

### Amplicons filtering, phylogenetic diversity, and taxonomic delineation

In total 159,995 raw rRNA sequences reads were obtained from the 32 sequenced samples. 101,362 reads were retained after quality filtering and clustered into 8,729 MOTUs. 907 MOTUs were assigned to eukaryotes other than foraminifera after comparison with the *PR²* database and were discarded (Supplementary Fig. 1, Supplementary Material 3). From the 7,822 MOTUs attributed to foraminifera, 176 occurred in at least three samples and had an abundance of at least ten amplicons. Seven MOTUs did not yield sufficiently long sequences to be interpreted in a phylogenetic framework and 14 MOTUs were identified as potential chimeras.

Phylogenetic inference including representative sequences of the retained 155 environmental MOTUs and 81 reference sequences of foraminifera resulted in 6 distinct but unsupported clades (Fig. 1A). Three clades were composed of reference sequences from microperforate (Fig. 1B), non-spinose (Fig. 1E), spinose and monolamellar (Fig. 1C) planktonic foraminifera and housed 58, 20 and 16 environmental MOTUs respectively. Two clades were composed of distantly related species of planktonic foraminifera characterized by significantly higher rates of rRNA substitution^38^ and are named “Long Branch I” (Fig. 1F) and “Long Branch II” (Fig. 1G). The “Long Branch I” clade was composed of reference sequences from two known morphospecies (*Globorotalia truncatulinoides* and *Turborotalita quinqueloba*) and housed two environmental MOTUs, whilst the “long-Branch II” clade included only one reference sequence from a known morphospecies (*Globorotalia menardii*) and 13 representative sequences of MOTUs. The last clade included representative sequences of benthic foraminifera plus the reference sequence for the basal non-spinose species *Globoquadrina conglomerata*. This clade, named “Basal”, was characterized by shorter branches and a lack of internal structure (Fig. 1D) and housed 42 environmental MOTUs.

A consistent barcode gap was observed between the “Intra-Genetic Type”, “Inter-Genetic Type” and “Inter-morphospecies” distances in the 6 clades (Supplementary Fig. 2). The intra- and inter-Genetic type distances were always statistically supported in all clades (Supplementary Material 1), validating the clustering conducted with ABGD. Although such a gap was identified in the “Basal” group between the successive levels, no molecular taxonomy exists for the reference sequences of this clade composed primarily by benthic lineages which are notorious for their high level of intragenomic variability that can be wrongly interpreted as genuine diversity^4^. We observed that the maximum distances observed within the ABGD defined “morphospecies” within this clade ranged from 4,47 to 6,51% which is compatible with the level of intraspecific variability observed in benthic foraminifera that can reach 35.2% in variable region^4^. Therefore, we chose a conservative interpretation of our results and considered that no genetic types was present below the “morphospecies” level of diversity within that clade (Fig. 1).

The two-step clustering of ABGD grouped the 155 representative sequences of environmental MOTUs into 46 genetic types belonging to 26 morphological species (Fig. 1). Amongst the 46 detected genetic types, 21 had been described previously and 7 consisted of new genetic types belonging to 5 known morphospecies (Fig. 1). The remaining genotypes clustered within 12 novel putative morphospecies identified by our approach. Five clustered among the Basal clade (*Basal A* to *Basal E*), two clustered among the Microperforate clade (*Micro A* and *Micro B*) and two clustered among Non-Spinose clade (*Non-Spinose A* and *Non-Spinose B*). Finally, we identified three putative morphospecies composed of six sub-lineages with unknown phylogenetic affinities as they clustered into the artificial “Long-Branch II” clade.

### Meta-reference attribution

The *Meta-Reference* database included representative sequences of the 155 environmental MOTUs together with 1,342 reference sequences issued from the PR² database and 1,272 issued from the PFR² database and possessed 361 unique taxonomic paths (Supplementary Material 3). After re-assignment of the dataset by the meta-reference, 6,010 out of the 7,822 MOTUs representing 88,734 reads (94.5% of the foraminiferal amplicons) presented a similarity with reference sequences of 95% or more (Supplementary Fig. 3). Among those, we identified 230 rare MOTUs with a high similarity with 15 taxa present only in PR² or PFR² databases (*Turborotalita quinqueloba* types I and II, *Globigerinoides ruber* type I, *Globigerinoides elongatus, Pulleniatina obliquiloculata* type II, *Neogloboquadrina sp*., *Neogloboquadrina incompta* type I*, Globorotalia tumida, Globorotalia truncatulinoides* type III, *Globorotalia scitula*, *Hastigerina pelagica* type II and *Globigerinita glutinata* type II, *Cibicides refulgens*, *Globocassidulina sp*. and *Nonion sp*.) that were not considered in the initial analysis. As a result, the final retained dataset for ecological inferences was composed of 69 robustly defined genetic types belonging to 41 morphospecies (Fig. 2).

### Ecological inferences

Rarefaction curves performed on the ecological dataset showed that saturation was reached in six of the 8 stations and for the total dataset (Fig. 3). Overall, saturation was reached at a sequencing depth of 5,000 reads for individual stations and 25,000 reads for size fractions. A higher diversity level was encountered in the micro-plankton [20–180 µm] compared to the meso-plankton [180–2000 µm]. Interestingly, the saturation curves for the total dataset and all the samples recovered in surface waters were similar and offset from the saturation curve for the subsurface community, showing that nearly all encountered species were occurring at the surface.

**Figure 4:**
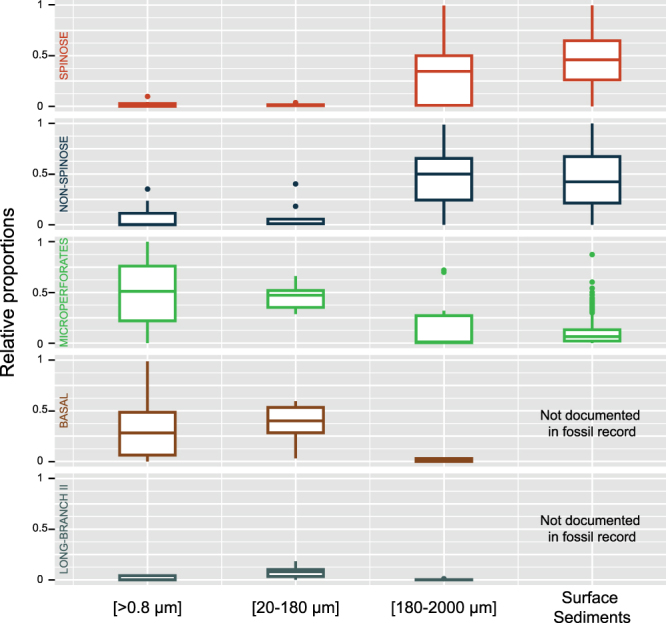
Relative contribution of the 5 clades in the three size classes and surface sediments (Data extracted from the MARGO database^30^). The box plot were drawn with ggplot2^67^ implemented in R^68^.

Finally, we assessed the relative abundance of reads from each of the 5 clades in the 3 size fractions. Variation in SSU rDNA copy number within single-cell can potentially bias the estimation of relative abundances in metagenomics surveys^57^. In benthic foraminifera, copy number can vary from 5,000–10,000 to 30,000–40,000 copies per single cell depending of the species^3^. There is no estimation to date of the number of SSU rDNA copies within single-cell of planktonic foraminifera, but we can reasonably assume that such bias exists as well. Therefore, we also compared the relative proportions of the census counts of foraminifera shells in surface sediments (Fig. 4). We found that the composition in the size fraction [180–2000 µm] roughly followed the relative proportions usually observed in surface sediments typically considering the size fraction >150 µm^30^. It shows that even if a bias in the SSU rDNA copies number is likely to exists, it does not modify the relative proportion between the major phylum of foraminifera. These assemblages were typically dominated by the Spinose and Non-Spinose clades. However, the size fraction [20–180 µm] was dominated by the Microperforate and Basal clades whilst the Spinose and Non-spinose clades were in low abundance. The size fraction [>0.8 µm] returns essentially the same result as the size fraction [20–180 µm] suggesting that the planktonic foraminifera community is in majority dominated by clades occupying small size fractions. The Long-Branch II clade was consistently restricted to the [20–180 µm] and [>0.8 µm] size fractions, implying that its constitutive species are small.

## Discussion

The extent of microbial diversity in the ocean has been a matter of controversy^58^. Our investigation of the diversity in planktonic foraminifera contributes to the resolution of this controversy by focusing on a clade with fully resolved classical taxonomy linked to barcodes. The target group is among the most extensively studied and character-rich groups of eukaryotic plankton, and after 200 years of taxonomy and 20 years of single-cell barcoding, its diversity appears modest and finite. Here we evaluate the extent of planktonic foraminifera diversity through metabarcoding of environmental samples, covering the vertical and latitudinal habitat of the group and all size classes where foraminifera cells are likely to occur.

Following a rigorous method of attribution and taxa delineation, we show that the metabarcoding results are largely congruent with previous taxonomy knowledge. Amongst the 69 lineages detected in the environmental metabarcode, 50 could be attributed to 28 morphospecies for which reference sequences are available in public databases (Fig. 2). Only seven of those genotypes have not been previously detected by single cell surveys (Fig. 2). Seven genotypes unrelated to any known morphospecies could be attributed to known morphogroups and clustered into four putative morphospecies (*Micro A*, *Micro B*, *Non-Spinose A*, *Non-Spinose B*; Figs 1 and 2). These could correspond to classical morphospecies absent from reference databases such as the Tenuitelids for the Microperforates or the Globorotalids for the Non-spinose. The 12 remaining lineages could not be attributed to any known morphogroup and were assigned to two artificial categories. These lineages occurred in the [20–180 µm] size fraction, which is usually not considered in micropaleontology^59^. Indeed, micropaleontologists classically work with specimen >150 µm because they are easier to handle, can be studied under a stereomicroscope and have more calcite weight for geochemical analyses^60^. The barcoding effort that led to the construction of the PFR² database^35^ was targeted on iconic morphospecies of planktonic foraminifera used in paleoceanography. The largest specimens more likely to yield enough DNA to be successfully PCR amplified were selected^61^. Therefore, it is logical that these 12 unknown genotypes are absent from the current databases because no barcoding effort has been directed so far on the size fraction where they occur. It is likely that some of those 12 lineages, structured into 8 putative morphospecies, could belong to described, but unsequenced morphospecies, such as *Orcadia riedeli*, *Dentigloborotalia* *anfracta* or *Berggrenia pumilio* which are usually small (Hemleben^31^). Indeed, the non-spinose morphospecies *Globoquadrina conglomerata* characterized by a slow rate of evolution clustered within the “Basal” clade and the three fast evolving morphospecies *Globorotalia truncatulinoides, Globorotalia menardii* and *Turborotalita quinqueloba* clustered into artificial groups (Fig. 1). Therefore, it is possible that these environmental genotypes match the documented morphological diversity, although they cluster outside of their “home” clades.

At the same time, we note that several abundant morphospecies in the sampled environment such as *Trilobatus sacculifer* and *Globigerinoides ruber* were rare in the molecular dataset, which indicates the existence of a PCR bias due to preferential amplification. This illustrates that metabarcoding, even if powerful, can still be partly blind^62^ and that we cannot entirely exclude that unknown and ecologically relevant groups could have also been missed during the amplification. In addition, we followed a stringent path to build our ecological dataset. We chose to retain only the most abundant MOTUs as a first step to build a taxonomic framework aligned with existing databases (our so called *meta-reference*) and to re-assign the whole dataset as a second step. By doing that we retained ~78% of the MOTUs diversity and ~95% of the dataset volume. Importantly, only after this step, MOTUs belonging to 14 morphospecies present in the reference database but rejected by our initial iteration were retained. Therefore, the same may apply to yet undocumented morphospecies represented by low abundance MOTUs in the dataset.

Despite these factors that could under-estimate genuine diversity, rarefaction curves indicated that a near-saturation level of diversity was reached for the total dataset and at all stations (Fig. 3) except for station 67 where half of the reads were non-foraminifera (Fig. 2C). Even if the biogeographic range covered in our study is limited, our results strongly suggest that the diversity in planktonic foraminifera is finite and in the range of what has been estimated through single-cell genetic surveys^50^. We detected up to four genetic types per morphospecies in our analysis, whereas up to seven were detected with single-cell genetic surveys^50^. When excluding taxa with a modest sampling coverage, single-cell genetic surveys suggest that the morphological taxonomy underestimate genuine diversity by a factor of three on average^50^. Assuming that only ~50 forminifera morophotaxa exist after two centuries of taxonomical efforts^31^, we can reasonably think that only a few hundred and not thousands of planktonic foraminifera biological species exist.

One striking result of our study is the clear difference in community composition among the different size fractions (Fig. 4). We observed than the Microperforate and Basal clades dominated the size fraction [20–180 µm] and [>0.8 µm] whilst the community composition of the size fraction [180–2000 µm] matched with the surface sediments counts (Fig. 4). The later indicates that the dominance of the Microperforate and Basal clades in the smaller size fractions is not an artifact induced by preferential amplification but a genuine pattern. It appears that the planktonic foraminiferal community in small size fraction consist mostly of poorly characterized or unidentified lineages, which have so far received little attention from taxonomists.

## Conclusion

The Tara metabarcoding survey of eukaryotic plankton diversity estimated the global diversity in the sunlit ocean at ~150,000 MOTUs, which is one order of magnitude higher than the 11,200 cataloged morphospecies described by traditional taxonomy^10^. Our results suggest that the implied unknown diversity might not be equally distributed among all planktonic lineages. Whilst some plankton groups are hyperdiverse such as the Diplonemids^63^, our results indicate that at least among the planktonic foraminifera this diversity is limited and not too far from estimates based on classical taxonomy and single-cell barcoding efforts. The case of planktonic foraminifera underlines the crucial role of coverage in reference databases used for the interpretation of metabarcoding data. Our survey shows that the diversity of planktonic foraminifera is finite but also that a considerable part of its biomass, resulting in a flux of at least 25–100 Tg carbon/year to the sea floor^64^, is made of small size taxa that received limited taxonomic attention.

### Data availability

The raw sequence data can be downloaded from the European Nucleotide Archive under BioProject PRJEB23355 (https://www.ebi.ac.uk/ena/data/view/PRJEB23355).

## Electronic supplementary material

Supplementary information

Supplementary Dataset 1

Supplementary Dataset 2

Supplementary Dataset 3

Supplementary Dataset 4
