# Supplementary material for: Surface ocean metabarcoding confirms limited diversity in planktonic foraminifera but reveals unknown hyper-abundant lineages
Source: Sci Rep. 2018 Feb 7;8:2539. doi: 10.1038/s41598-018-20833-z (PMC5803224; doi:10.1038/s41598-018-20833-z)
Supplement: Supplementary file 1 — Supplementary information [file 41598_2018_20833_MOESM1_ESM.pdf]

Supporting information for:

Surface ocean metabarcoding confirms limited diversity in planktonic foraminifera but reveals unknown hyper-abundant lineages

Raphaël Morard[1,2,*], Marie-José Garet-Delmas[2], Frédéric Mahé[3], Sarah Romac[2], Julie Poulain[4], Michal Kucera[1] and Colomban de Vargas[2]

[1]MARUM Center for Marine Environmental Sciences, University of Bremen, Leobener Strasse, 28359 Bremen, Germany

[2]Station Biologique de Roscoff, UMR7144, Sorbonne Universités UPMC Université Paris 06 & CNRS, Place Georges Teissier, 29680 Roscoff, France

[3]CIRAD, UMR LSTM, F-34398, Montpellier, France

[4]CEA - GENOSCOPE- Institut François Jacob, 2 rue Gaston Crémieux, 91057 Evry, France.

**corresponding author: rmorard@marum.de*

Supplementary Figure 1

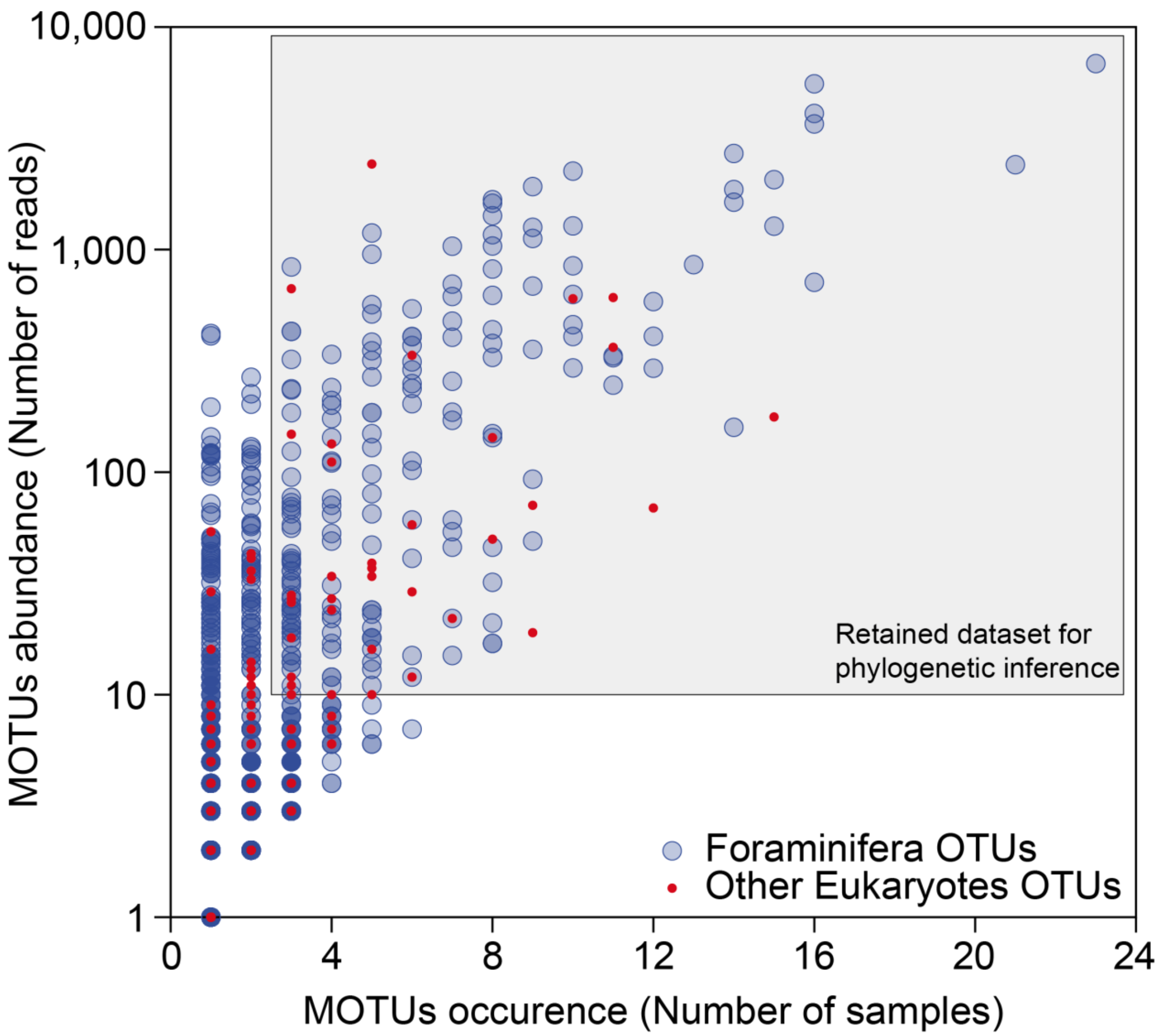

Supplementary figure 1. Occurrence versus abundance plot of the 8,471 rDNA MOTUs assembled from the dataset. The colors indicate their taxonomic attribution after comparison to the *PR²* database (Guillou et al., 2012). The rectangle encloses the MOTUs considered to characterize the foraminiferal diversity within the dataset (Fig. 1).

Supplementary Figure 2

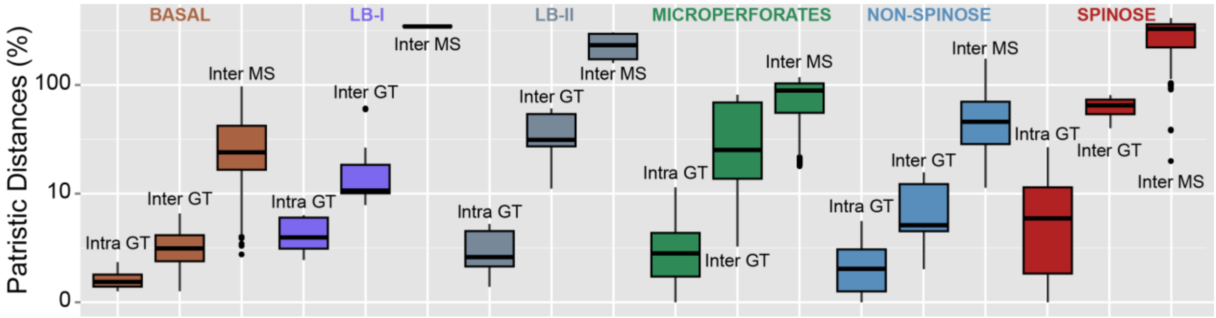

Supplementary figure 2. Distribution of the patristic distances for the Intra Genetic Type (Intra GT), Inter Genetic Type (Inter GT) and Inter Morphospecies (Inter MS) for each of the six clades (Fig. 1) drawn with ggplot2[58] implemented in R (R Development Core Team 2014).

Supplementary Figure 3

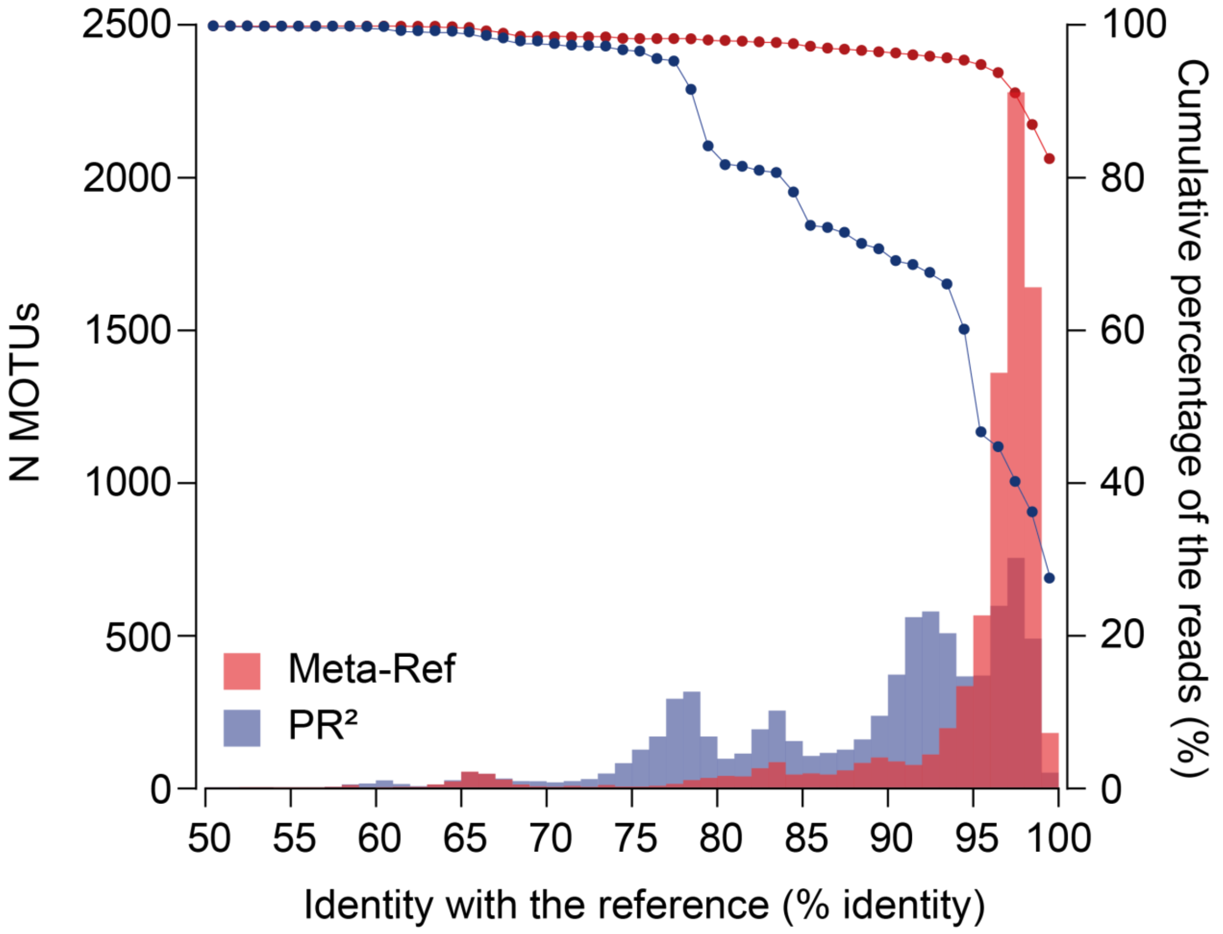

Supplementary figure 3. Genetic identity of the environmental MOTUs to PR² and the meta-reference databases (see Material & Methods section). The histograms show the identity level of the MOTUs with the PR² database and the meta-reference. The connected dots show the cumulative percentage of the dataset volume (reads).

Supplementary Table 1

Clade	Nb. of Intra Genetic Type distances	Nb. of Inter Genetic Type distances	Kolmogorov-Smirnov test		Mann-Whitney test	
			p	p(permutation)	p	p(permutation)
Basal	301	1469	6,55E-221	0,0001	6,58E-165	0,0001
Long Branch I	11	18	4,09E-07	0,0001	9,54E-06	0,0001
Long Branch II	38	58	1,42E-21	0,0001	1,55E-16	0,0001
Microperforates	535	1586	1,31E-129	0,0001	4,41E-153	0,0001
Non-Spinose	39	571	4,79E-28	0,0001	1,62E-23	0,0001
Spinose	87	648	1,91E-68	0,0001	5,57E-51	0,0001

Supplementary Table 1. Results of the Kolmogorov-Smirnov and Mann-Whitney tests for patristic distance comparisons.
